# Living with Ghosts: How Physical Traces of the Past Shape Cultural Trauma in Chinatowns

**DOI:** 10.1177/00031224261422414

**Published:** 2026-03-13

**Authors:** Matt Patterson, Henry Tsang, Bryan Kuk, Weiqi Li, Mojtaba Rostami

**Affiliations:** a University of Calgary; b Athabasca University; c University of British Columbia

**Keywords:** cultural trauma, Chinatowns, collective memory, space and place, materiality

## Abstract

Cultural trauma refers to how past experiences of harm can fundamentally transform a community’s shared identity, potentially generating feelings of solidarity and providing communities with a sense of common purpose. This article examines the role of cultural trauma in motivating and guiding ongoing efforts to preserve historic Chinatowns in Canada and the United States in the face of contemporary challenges such as gentrification. We demonstrate how activists understand themselves to be continuing a struggle against anti-Chinese racism that extends back to the nineteenth century. Explaining the contemporary salience of trauma, we conceptualize Chinatowns as “cultural reservoirs” that have accumulated physical traces of past harms. These traces serve as iconic representations of trauma: haunting reminders of the tenuous place of Chinatowns in North American cities. We identify three types of icons produced by distinct material processes: stubborn, entropic, and remedial. By identifying the importance of the physical environment in the trauma process, we reconcile a realist focus on historical events themselves with a constructivist account of their subsequent incorporation into collective memory. Cultural reservoirs do not determine how contemporary communities will remember their past, but the physical traces they preserve create experiences and situations in which trauma narratives seem intuitive and salient to contemporary communities.

I walk

on earth

above the bones

of a multitude

of golden mountain men

searching for scraps

of memory

— Excerpt from “old chinese cemetery kamloops july 1977” in *Chinatown Ghosts* by Jim Wong-Chu (2018)

The words of the late Vancouver-based poet Jim Wong-Chu interrogate issues of identity and belonging for Chinese Canadians living in a country with a well-documented history of anti-Chinese racism (P. Li 1998). The title of Wong-Chu’s poetry collection, *Chinatown Ghosts*, evokes the idea of this troubling past lingering in the landscape and affecting the lives of contemporary generations. As such, his work exemplifies what Alexander (2004:1) calls “cultural trauma,” defined as a situation where “members of a collectivity feel they have been subjected to a horrendous event that leaves indelible marks upon their group consciousness, marking their memories forever and changing their future identity in fundamental and irrevocable ways.” Along with the harm of the events themselves, as collective memory, cultural traumas can also generate social solidarity, as communities transform individual suffering into a collective responsibility (Alexander 2004:27). While tied to the past, collective memories provide a common conceptual framework through which communities understand their present circumstances and work toward future goals.

Our initial aim here is to demonstrate the role of cultural trauma in motivating and guiding ongoing efforts to preserve historic Chinatowns across the United States and Canada. We draw on interviews and fieldwork with “Save Chinatown” activists and dozens of planning documents. Established in the late-nineteenth and early-twentieth centuries, historic Chinatowns have proven remarkably resilient, surviving anti-Chinese riots, Chinese exclusion and head tax laws, and postwar slum clearance programs. Today, these neighborhoods face new challenges, including gentrification (Hom 2024), the anti-Asian racism and economic shocks of the COVID-19 pandemic (Chen and Wu 2025), and the fact that Chinatowns play an ever smaller role in migration processes and in the lives of Chinese Americans/Canadians more generally (Li and Li 2011; Liang et al. 2018). As we will argue, cultural trauma provides an explanation for why these neighborhoods continue to matter to the people fighting to preserve them.

Identifying the importance of cultural trauma for Chinatown activists raises a second, more fundamental question. How do harmful events of the past come to resonate with subsequent generations who did not experience these events directly? Within cultural trauma research, this question has been a sticking point between realists, who study the direct effects of historic events themselves (e.g., Erikson 1976), and constructivists who focus on the subsequent production of collective memory (e.g., Alexander 2004; Eyerman 2001). In reconciling these perspectives, we take inspiration from Jim Wong-Chu, who reminds us that historic harms not only live on in minds or even words; rather, historic harms leave physical traces in the landscape that can haunt the living like ghosts. Following this insight, we present a “materialist” approach to cultural trauma that focuses on how these physical traces mediate the relationship between past events and contemporary memory. Physical traces do not determine how or if an event will be incorporated into collective memory as a trauma, but they can become powerful, independent factors in commemoration.

To explain the salience of trauma narratives within these communities, and drawing on insights from research on place and materiality, we demonstrate how Chinatowns serve as “cultural reservoirs” that have accumulated the traces of historic harms. These traces are imbued with “iconic power”: they act as powerful representations of trauma (Bartmanski and Alexander 2012). We identify three types of icons, each created by unique material processes. “Stubborn” icons are produced during periods of harm and remain stuck in the landscape over generations. “Entropic” icons are the opposite, becoming iconic when they break down or disappear. Finally, “remedial” icons are produced when affected communities transform physical remnants of past harm into purposely-designed memorials. A “cultural reservoir” is a place that has accumulated many such traces, creating a level of coherence or “mnemonic relatedness” (Suttles 1984:294) to past harm that is greater than the sum of its parts. People in Chinatown are routinely confronted by trauma icons. This experience is more diffuse and mundane than purposefully-designed memorial sites (Simko 2020), and yet more uniquely powerful in evoking feelings of the past than is captured by more general concepts such as “place character” (Molotch, Freudenburg, and Paulsen 2000) or “scenes” (Silver and Clark 2016) that describe how people intuit meanings from all places. Overall, cultural reservoirs help us understand the role that past events play in shaping the material conditions of commemoration.

Our argument proceeds in five parts. First, we discuss the realist/constructivist divide in cultural trauma and outline a materialist synthesis. Second, we review the history of Chinatown as an object of sociological study and summarize the research project on which this article draws. Third, we demonstrate the centrality of trauma narratives in Chinatown preservation movements. Fourth, we introduce and provide evidence for the three types of trauma icons found within Chinatowns. Finally, we encapsulate our findings by theorizing Chinatown as a cultural reservoir and relate this concept to the larger literatures on cultural trauma, place, and ethnic enclaves.

## Explaining Cultural Trauma

Initially referring to bodily harm, psychologists adopted the term “trauma” as a metaphor to describe prolonged mental harm (Simko and Olick 2020:653). The DSM-5 identifies a series of trauma-related disorders, including posttraumatic stress disorder (PTSD), which is defined as a condition where previous “exposure to actual or threatened” harm produces negative psychological and behavioral symptoms, including “recurrent, involuntary, and intrusive distressing memories of the traumatic event(s)” (American Psychiatric Association 2013:271). In Durkheimian fashion, sociologists have redefined trauma as a group-level phenomenon related to a breakdown of culture or social structure. These theories then bifurcate into what we can call “realist” and “constructivist” approaches.

### Realist Theories

Realist accounts of trauma are exemplified by Erikson (1976:154), who distinguished “individual trauma,” which he defined in line with PTSD, from “collective trauma” caused by “a blow to the basic tissues of social life that damages the bonds attaching people together and impairs the prevailing sense of community.” To illustrate, Erikson (1976:154) cites communities displaced by slum clearance projects “whose feelings of well-being begin to deteriorate,” not because of direct exposure to physical harm, but rather “because the surrounding community is stripped away and can no longer supply a base of support.”

Psychologists themselves increasingly distinguish individual-level PTSD from collective or “historical trauma” (HT), which “focuses attention on the role of dominant groups in systematically generating and perpetuating a collective trauma that continues for an extended time [and leads] to multigenerational disruptions in the well-being of the targeted group” (Nagata, Kim, and Gone 2024:2). Nagata and colleagues (2024:15) identify Asian Americans as fitting many of the criteria of HT, citing extensive research done on the intergenerational transmission of trauma connected to Japanese internment during WWII, and refugee migration out of Southeast Asia during the Vietnam War. Although less studied in HT, the history of the Chinese diaspora in Canada and the United States is similarly punctuated by episodes of collective harm (P. Li 1998; Tong 2000), including passage of the U.S. Chinese Exclusion Act (1882 to 1942) and the Canadian Chinese Head Tax (1885 to 1923) and Exclusion Act (1923 to 1947), which severely limited Chinese immigration, resulting in the destruction of communities and separation of families.

Beyond academia, realist understandings of trauma are implicit in much of the public discourse and institutions established to address past atrocities. Truth and Reconciliation Commissions established in countries such as Chile, South Africa, and Canada were designed to produce authoritative accounts of past harms, identifying the type of harm committed, the victims, and those responsible (Alexander 2004:12–15). State apologies serve a similar function, including those offered by the Canadian Parliament in 2006, and the U.S. Congress in 2011 and 2012 for the aforementioned Chinese exclusion laws.

### Constructivism and Its Critics

Associated with the “Strong Program” in cultural sociology, constructivists focus less on establishing authoritative accounts of actual harm in favor of studying the “trauma process” through which realist accounts get developed and accepted (Alexander 2004:11). Eyerman’s (2001:23–24) study of slavery and African American identity, for example, situates the “roots and routes of cultural trauma” two decades after emancipation as a “new black middle class emerging after the civil war . . . [attempted to counter] the image of blacks being put forward by whites.” Eyerman (2001:2) notes that historic events may be “the significant ‘cause’” of cultural trauma, but they are only the start of “a process which requires time, as well as mediation and representation.”

More forcefully, Alexander (2004:10) accuses realists of a “naturalistic fallacy” in which memory is treated as a reflection of an unambiguous past: “Events are one thing,” he argues, “representation of these events are quite another.” Past harms are not necessarily remembered as such, particularly when powerful people have an interest in suppressing them. Even victims may repress their own experiences. According to this perspective, if a harmful event is lost to history, or if it fails to sufficiently resonate with subsequent generations, it cannot function as a cultural trauma.

For constructivists, trauma is sociologically important because it helps establish and extend solidarity and collective identity in the present, as well as envisioning possible futures—a process Alexander (2004:23) calls “working through” trauma. In Eyerman’s study, the trauma of slavery formed the basis of a new “African American” identity. Similarly, Abrutyn (2015:128) argues that cultural trauma helps explain the longevity of Jewish identity; he notes that trauma can provide “charter narratives” that groups use to understand who they are and where they come from.

Constructivists have attracted their own critics. Joas (2005:371) argues that Alexander “does not make a clear distinction between psychological and social consequences of traumas on the one hand and the social construction of cultural memory and a phenomenon called ‘trauma’ on the other hand.” Joas (2005:373, 369) further suggests that Alexander’s “‘culturalism’ should be checked by an ‘experientialism,’” and focusing on “the dimension of social construction [does not provide] a way around the question of what has ‘really’ happened, who has ‘really’ been affected by an event.” Joas (2005:371) suggests the term “trauma” should be reserved for actual experiences of harm (e.g., PTSD) and distinguished from collective memory.

In their efforts to distinguish themselves from realism, constructivists have arguably undertheorized the connection between actual experiences of harm and their subsequent incorporation into collective memory as cultural trauma. However, constructivism itself does not close off the possibility of bridging this gap. To justify the term “trauma” while also avoiding the “naturalistic fallacy,” we must show two things: First, that the historic events in question fundamentally altered the conditions under which a community understands itself. Second, that contemporary generations continue to experience strong negative emotions associated with those events (Smelser 2004; Woods 2019), and these emotions inspire fear of future harm. In other words, we must show that trauma narratives have a high level of practical and emotional “resonance” (McDonnell, Bail, and Tavory 2017). Establishing these factors allows us to balance what Joas calls a “culturalism” that attends to the creation and deployment of trauma narratives, with an “experientialism” that demonstrates how historic harms disrupt the lives of a community, sometimes across generations.

### The Materiality of Cultural Trauma

One way to bridge this gap is to focus on how past events shape the material conditions under which memories are developed and articulated. Outside sociology, architects, artists, and placemakers have explored how past harms create “traumascapes” by shaping the physical environment (Courage and McKeown 2024; Tumarkin 2019). Sociologists themselves have long been interested in how the physical environment connects the past to collective memory. Suttles (1984) described the “cumulative texture” of collective representations that amass over time within specific locations (e.g., plaques, road names, sculptures). Molotch and colleagues (2000:793) expanded this idea, arguing that “place character” is not limited to explicit mnemonic objects, but created by the whole “array of physical and social elements” clustered in a locale. Place character can in turn influence actors’ dispositions (e.g., feelings of fear, comfort, excitement, solemnity), as well as higher-level forms of sense-making, such as the adoption of specific narratives or evaluative frames (Babon 2006; Patterson 2016). Even personal identity can be shaped when the people, objects, and situations within a given place continually “summon” people to perform certain roles in their daily lives (Tavory 2016; see also Brown-Saracino 2018).

Physical places also feature in constructivist accounts of trauma and collective memory. Alexander (2012:88–91) argues that physical memorials “crystaliz[e] collective sentiment” around trauma by creating experiences that “provide opportunities for contemporaries, now so far removed from the original scene, powerfully to reexperience it.” Simko (2020:56) similarly argues that memorial sites are “one of the most important vehicles for conferring recognition upon victims.” These studies primarily focus on the contingent, creative process by which “memorial entrepreneurs” (Jordan 2006:11) construct narratives and assemble political support to build memorials (Jordan 2006; Simko 2020; Wagner-Pacifici and Schwartz 1991).

Memorial entrepreneurs play an essential role in constructing trauma narratives and collective memories, but their work does not take place on a “blank slate” (Jordan 2006:23). Rather, the process of commemoration occurs within a “figured” landscape “inflected with cultural ideals and conveying a reality that seems inevitable, natural, or true” (Mukerji 2010:404). In other words, the physical landscape can influence the perceived authenticity and resonance of narratives. This is especially true in places with “mnemonic relatedness” (Suttles 1984:294), where the same meanings are continually reinforced by various objects and experiences. Jordan (2006:14) notes how people often speak of an “invisible aura” that emanates from locations associated with great harms. Similar to Jim Wong-Chu, some sociologists have evoked the idea of “ghosts” to describe how our experience of place is sometimes shaped by a feeling of “presence” for people and things that are no longer physically there (Bell 1997; Brown-Saracino 2021:1021).

Understanding these associations does not require us to believe in spirits, but it does lead us to further investigate the mechanisms through which the locations of past harms can influence the conditions of their commemoration as traumas. To do this, we draw on recent work in the sociology of materiality, which challenges the constructivist tendency to view material objects as passive receptacles onto which humans project cultural meanings (Jerolmack and Tavory 2014:66). Instead, this work investigates how the material qualities of objects actively shape cultural processes, including the production and interpretation of meanings, and provides a toolkit of concepts, such as “cultural entropy” (McDonnell 2016), “embodied metaphor” (Winchester 2016), and “iconicity” (Bartmanski and Alexander 2012), that are useful in explaining the connection between the material landscape and commemoration.

## The Sociology of Chinatowns

Chinatowns are important sites for investigating how cultural connections are forged across generations, particularly connections related to trauma. The neighborhoods examined in this article trace their origins to the late-nineteenth century as Chinese people began migrating through the newly-colonized port of Hong Kong to the United States and Canada, then known as “Gold Mountain” (*Gam Saan*, 金山). Both countries eagerly exploited Chinese labor to complete their respective transcontinental railways. However, Chinese migrants were met with hostility, legal sanctions, and even violence, which Pfaelzer (2008) characterizes as a campaign of “ethnic cleansing.” These events include anti-Chinese riots in cities such as Los Angeles (1871), Vancouver (1887), and Calgary (1892), and restrictions on civil and economic rights enforced by local and state/provincial governments. With the completion of the railways, both countries passed their head tax and exclusion laws, ushering in the “Exclusion era” (1880s to 1940s). Despite these restrictions, tens of thousands of Chinese people remained in North America—mostly male “bachelors” who were legally and financially unable to bring over their families. Often restricted in where they could live and operate businesses, they established segregated enclaves (“Chinatowns”) that provided safe residential spaces, employment opportunities, and social and cultural support (Zhou 1992:33–35).

For good or ill, Chinatowns have long attracted the attention of outsiders, including sociologists who have looked to these neighborhoods to understand the intersection of migration, race and ethnicity, community formation, social disorder, and segregation. The size and richness of this research arguably make the “Sociology of Chinatown” its own unique subfield. Tracing the history of this literature gives us insight not only into the neighborhoods themselves, but also the discipline of sociology more generally.

**Figure 1.**
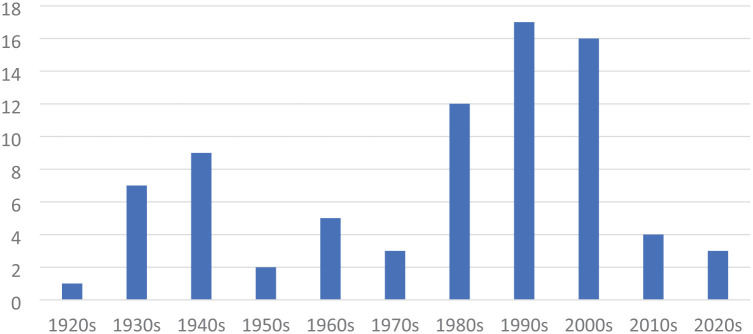
The Number of Articles Containing the Term “Chinatown” Published per Decade in the *American Sociological Review*, *American Journal of Sociology*, and *Social Forces*

Figure 1 tracks the number of articles that mention “Chinatown” in the *American Sociological Review*, *American Journal of Sociology*, and *Social Forces*. The figure reveals two waves when Chinatowns became frequent topics of general sociological discussion. The first wave corresponds to the Chicago School-inspired ecological work of scholars such as Rose Hum Lee (1949), Beulah Ong Kwoh (1947), Clarence Glick (1942), and Paul C. P. Siu (1952).^1^ The Chinatown they encountered was the “bachelor society” in economic and demographic decline in the final years of the Exclusion era. Some interpreted this trend as evidence for the Chicago School’s theory of spatial assimilation (e.g., Lee 1949).

Mirroring the decline of the Chicago School itself, sociological interest in Chinatowns waned in the postwar decades. Chinese exclusion was repealed in 1942 in the United States and in 1947 in Canada (under pressure from Chinese American/Canadian WWII veterans, who demanded citizenship rights). Despite this achievement, Chinatowns faced a new challenge in the form of “slum clearance” or “urban renewal” campaigns that sought to demolish aging inner-city neighborhoods to make way for expressways and other infrastructure (Vitiello and Blickenderfer 2020). The result was the disappearance of many Chinatowns, especially in smaller towns in the west (Lai 1988). In larger cities, urban renewal provoked a political awakening. “Save Chinatown” opposition movements arose, halting or curtailing the demolition of their neighborhoods (Chan 2011; Lin 1998; Wilson 2015).

Renewed sociological interest beginning in the 1980s coincided with a revitalization period for many Chinatowns (Lai 1988:4–8). “Save Chinatown” movements were successful in redefining their neighborhoods in the public imagination from social problems to cultural assets. Urban planners and policymakers adopted new attitudes toward the value of multiculturalism and diversity (Fainstein 2005). Finally, following the 1940s repeal of exclusion, the United States and Canada gradually liberalized their immigration laws, passing major reforms in the 1960s and 1970s that removed de facto discrimination against non-Europeans. These reforms, combined with the East Asia economic boom, resulted in an influx of people and capital back into historic Chinatowns by the 1980s (Zhou 1992).

This trend attracted a new generation of migration scholars studying immigrant economies and settlement patterns (Portes and Sensenbrenner 1993; Sanders and Nee 1987; Zhou and Logan 1989). Not captured in Figure 1 are several important Chinatown books also published in this period (Anderson 1991; Kwong 1987; Lai 1988; Lin 1998; Wong 1982; Zhou 1992). In contrast to the Chicago School, this generation drew distinctions between cultural assimilation and socioeconomic mobility and was more attentive to power and labor market dynamics.

Despite their differences, both generations viewed Chinatowns through an “enclave epistemology” that connects ethnic clustering to migration processes (Ghaziani 2019:5). Today, however, historic Chinatowns play an increasingly marginal role in migration, as Asian immigrants settle in the suburbs and smaller cities (Fong, Matsuo, and Wilkes 2007; W. Li 1998; Liang et al. 2018). Moreover, the Chinese diaspora is larger, more diverse, and more geographically distributed than ever before (Tran 2024), meaning that historic Chinatowns are only one relatively niche island within a larger Chinese “cultural archipelago” (Ghaziani 2019). Chinatowns no longer hold a monopoly as the place “where we go to be Chinese,” to quote journalist Bonnie Tsui (2009:2).

The declining significance of Chinatowns to Asian American life corresponds to the drop-off in sociological attention in the 2010s. Today, most Chinatown research focuses on gentrification or cultural preservation (Acolin and Vitiello 2018; Lin 2010; Naram 2017; Sotomayor and Zheng 2024; Sze 2010). Several studies have examined the rise of new “Save Chinatown” movements, reminiscent of the 1960s (Hom 2024; Mahieus and McCann 2023; Wong 2019). As these studies demonstrate, despite their marginalized status, Chinatowns continue to resonate strongly with contemporary generations who differ from the working-class “bachelor society” that established them in the nineteenth century. A few studies explicitly identify the role of cultural trauma in Chinatown activism, including Lin (2010) who discusses trauma in ethnic placemaking initiatives, and Zheng (2021) who demonstrates how Chinatown activists in Toronto cite family members who paid the infamous head tax as a way of proving their credibility.

Building on this literature, we return to a broader question at the heart of the two previous waves: why do Chinatowns resonate for the communities that sustain them? In the following sections, we demonstrate how cultural trauma helps us understand the motivations of “Save Chinatown” movements. We then explain why trauma narratives are so ubiquitous in Chinatowns, identifying the role of the physical environment in making past episodes of harm salient to contemporary generations.

## Methods

### Data Sources

This article draws on a mixed-method study of the emergence, motivations, and strategies of Chinatown preservation campaigns across Canada and the United States, which began in 2019 in support of a campaign in Calgary, Alberta, and involves a team of social scientists and architects. We conducted the initial 44 semi-structured interviews with Calgary-based Chinatown “placemakers,” defined as people who were actively involved in shaping the physical, social, and cultural character of the neighborhood for the purposes of preserving it as a Chinatown. Placemakers included community activists, leaders of community associations, business owners, artists, architects, and urban planners. We were interested in (1) their personal ties to the neighborhood, and (2) how they conceptualized Chinatown’s identity in their placemaking work.

Additionally, our team attended or participated in a variety of community meetings, advisory groups, and placemaking initiatives within the community, such as designing a local seniors center and advising on an exhibition on Chinese Canadian history. These experiences allowed us to triangulate what placemakers reported in interviews with how they engaged directly in placemaking practices. To fact-check interviews and expand the temporal range of our research, we also analyzed relevant archival documents, including neighborhood plans, news reports, and various public datasets (e.g., censuses, business licences, development permits, crime and traffic incidents).

We expanded the research beyond Calgary by analyzing Chinatown planning documents published between 1970 and 2024 in 15 cities: Boston, Chicago, Edmonton, Honolulu, Los Angeles, Montreal, New York City, Philadelphia, Portland, San Francisco, Seattle, Toronto, Vancouver, Washington DC, and Winnipeg. Combined with Calgary, this included 83 documents totaling over 5,000 pages. These plans are a common tool of “Save Chinatown” movements, produced directly by Chinatown-based organizations or by local governments (often in response to community pressure). They set out rules, guidelines, and goals for development and public investments and identify community concerns and problems. More generally, these plans present legitimated narratives about Chinatowns’ origins, core identity, and vision for the future. These plans allowed us to test our initial findings from Calgary in other Chinatowns.

We conducted 24 additional interviews and in-person field observations in Chinatowns in Chicago, Edmonton, Philadelphia, Toronto, and Vancouver. Our 68 total interviews were conducted in English (46), Mandarin (18), and Cantonese (3),^2^ based on participant preference. Our participants ranged in age from 18 to 90, averaging around 50. Female and male participants were roughly even, as were those born in North America and in Asia. The vast majority identified as ethnically Chinese. Most had postsecondary educations. Most participants requested that we use their real names. Any use of pseudonyms is indicated below.

### Analytic Strategy

The demographics of our participants reflect our decision to study placemakers rather than residents. This was strategic; we were interested in people who felt strongly enough about these neighborhoods as “Chinatowns” that they mobilized to preserve them as such. Nonetheless, our data do not capture all (or even a majority of) understandings and ways of relating to these neighborhoods. As previous studies demonstrate, people living in ethnic enclaves often disagree on their identities and boundaries (Brown-Saracino 2009; Hwang 2016). We encountered several residents who did not consider themselves to be living in a Chinatown, or at least were not concerned about preserving that identity. This group is not the focus of our study. Our participants spent time regularly in Chinatown for work (paid or volunteer) or leisure, but only a minority lived or owned property in the neighborhoods. Urban sociologists traditionally understand social investment in place as deriving either from residential “use-value” or commercial “exchange-value” (e.g., Logan and Molotch 1987). That neither of these motivations neatly explain the work of Chinatown activists presented itself as a compelling sociological puzzle.

This puzzle led us to discern dominant “neighborhood narrative frames” (NNF) within the data, which Small (2009) argues can motivate local civic engagement. Small (2009:70) defines NNFs as “concrete sets of categories through which the neighborhood’s houses, streets, parks, population, location, families, murals, history, heritage, and institutions are made sense of and understood.” We began analysis by “open coding” a diffuse list of topics, ranging from concrete (“lack of parking”) to abstract (“authenticity”), followed by “fixed coding” a shorter, more coherent list of NNFs (Emerson, Fretz, and Shaw 1995). Following Small (2009), we were attentive to the discrete spatial reference points that people make, which reveal aspects of the built environment that are most salient to particular NNFs. As we completed this process, we were guided by a logic of “abduction,” meaning we recognized that the NNFs we observed would reflect the theoretical lens we applied to the data (Timmermans and Tavory 2012). As a result, we used an “alternative casing” strategy of revisiting our data from different theoretical perspectives, looking for “anomalies” or “puzzles” that create opportunities for theory creation.

Through this process, we came to see Chinatown preservation as a case of “cultural trauma.” In addition to helping us understand the motivations and self-conceptions of placemakers, adopting a cultural trauma perspective opened a new theoretical puzzle about how harmful events of the past shape the conditions of commemoration in the present.

## Trauma Narratives in Chinatowns

Cultural trauma was not initially part of our theoretical framework; we considered it only after accumulating observations that did not fit earlier theoretical frames. One of these observations was explicit use of the word “trauma” in interviews, casual conversations, and public events. For example, Teresa Tam, a Canadian-born artist in her early-30s, described “the privilege of having a whole section of the city [to] dump my traumas and have it dump trauma on me.” The term did not come up frequently (only in 7 of our 68 interviews) and was almost exclusively used by younger people. However, it was so discordant with our existing theoretical understandings of Chinatowns as vibrant consumer destinations, or places where immigrants go to find work or housing, that it stood out.

Operationalizing cultural trauma as any instance where harmful past events are discursively linked to ongoing preservation or placemaking work, it became clear that trauma narratives are ubiquitous in Chinatowns, even if the word “trauma” is not. We routinely encountered plaques, monuments, and written documents that tied the origin and identity of Chinatown to harmful past events, such as Chinese exclusion or urban renewal. Although we had no interview questions about trauma or even history, a majority of participants voiced some type of trauma narrative. This finding cut across generations, gender, and national origin.

Consider Qi (pseudonym), a community organizer in her 60s who immigrated to Calgary from Hong Kong in the 1970s. When asked about the importance of Chinatown, she replied:

[Chinatowns] remind us of our social past. Some people actually think that we chose to have a Chinatown, but it was not an option. It was not a cultural destination to begin with because of blatant discrimination. Part of that learning . . . is that this is already the third Chinatown. . . . Why is it that there is a third [Chinatown]? It’s because the community was forced to relocate twice. If we are not diligent enough to recognize it and protect this culturally significant place, then it will be forced to move again.

Here, Qi recounts a “charter narrative” (Abrutyn 2015:128) that ties the community’s origins to a past harm. This narrative, specific to Calgary, refers to the displacement of the Chinese community from two previous locations in the city.^3^ The first Chinatown was severely damaged in 1892 following an anti-Chinese riot (Burnett 2012). In the second Chinatown, landlords evicted the community to make way for railway expansion (Lai 1988:89). The third Chinatown was established further from the city center, on the banks of the flood-prone Bow River. Even in this location, Chinese entrepreneurs had to overcome local opposition in their attempt to purchase property. Versions of this narrative can be found in the opening pages of Calgary’s new “Chinatown Cultural Plan” and on display in the local Chinese Cultural Centre, among other places.

These events happened decades before Qi was born, but they are salient to her understanding of the neighborhood and her work as an activist. They also inspire fear that a similar harm may be revisited upon her community in the future, particularly as Calgary’s Chinatown faces the pressures of gentrification and developers look for new land to expand the neighboring central business district. Qi’s quotation also demonstrates how memories of past harms inspire an emotionally-informed sense of collective obligation toward the neighborhood. In this way, our interviews corroborated both cultural trauma theory and Small’s (2009) observation that NNFs can motivate civic engagement.

It was not just the ubiquity of trauma narratives in our interviews that caught our attention. Such narratives also occupied prominent positions in public discourse, including in the opening pages of Chinatown neighborhood plans. For example, the Philadelphia Chinatown Development Corporation opened its 2017 neighborhood plan by stating:

The legacy of [Chinatown’s] founding history, rooted in racial discrimination and injustice, still reverberates throughout the neighborhood’s built environment and social and economic relations, and is in many ways unchanged. In order to protect what we love about Chinatown, we must plan for the future with consideration of this legacy. (p. ii)

In addition to exemplifying a “charter narrative” tied to collective trauma, the quotation also demonstrates how these narratives connect past harms to the present situation (“in many ways unchanged”) and future goals (“we must plan for the future with consideration of this legacy”).

These narratives are also found in government-authored plans, including the 2023 Calgary “Chinatown Area Redevelopment Plan,” which begins by stating that “this Plan provides direction for further study, accountability and investment to work to correct issues resulting from systemic racism” (City of Calgary 2023:iv). This is not standard language for a City of Calgary planning document. Prior to the plan’s publication, a non-Chinese staff member working on it shared what he had learned:

The leaders of this community told us that there’s been a history that dates back to the 1800s of systemic racism: policies and regulations that put Chinese [people] and the Chinatown of Calgary at a disadvantage. And those have followed through right up to [the] present day. What they’ve told us is: “in order for you to truly understand what Chinatown is about, you need to look at the history.”

### Community Variation

In emphasizing the ubiquity of trauma narratives, it is important not to misrepresent the significant heterogeneity and political divisions within Chinatowns. Research on Chinatowns often documents intense struggles between different groups claiming to speak for the neighborhood (e.g., Leong 1995; Wilson 2015). Many participants voiced frustration over an inability to work together. Alan Tong, a Canadian-born Chinese man in his mid-40s who is involved in public safety initiatives in Calgary Chinatown, put it this way:

There is no united Chinese population here. It’s ridiculous! It’s such a fractious community. There’s dozens of community associations. You can have the Fujian Community Association, the Chao Chow Community Association, the association of people from Singapore, Malaysia, and Brunei, the Taiwanese population, the mainland population, the Hong Kong population. You put politics in here, too. . . . There’s no unity here!

In addition to these ethno-cultural differences, we also found generational differences, which tend to overlap with other social cleavages. Placemaking initiatives in Chinatown often bring together young, progressive artists and nonprofit workers with older immigrant business-owners with more conservative political views. This generational gap influenced how people spoke about trauma. Older participants sometimes complained that the younger generation did not appreciate the history of struggle. “They don’t understand the situation in the old days. We were not wealthy like nowadays,” noted Jack Yee, a 90-year-old who immigrated to Canada in the 1950s to join his father following the repeal of Chinese exclusion. Younger participants complained that the “old guard” (a common phrase) was unwilling to “work through” trauma, which made them suspicious of change.

Trauma narratives stood out among other NNFs because they more often bridged ethno-linguistic and generational divides. In fact, several of our participants mentioned explicitly that they felt connected to previous generations through a common struggle against racism and displacement. Mark (pseudonym), a Philadelphia-based activist in his 50s, spoke of being inspired by the generation who engaged in a decades-long fight against the Vine Street Expressway, which cuts through Chinatown and was eventually completed in the 1990s: “When the highway was going [in], they organized the community. So, there’s this feeling of indebtedness to them that has helped us sustain ourselves. I think the passion is just so strong.” Meanwhile, Brian, a member of the Calgary Chinatown Business Improvement Association, discussed how anti-Chinese racism during COVID-19 helped him identify with and understand previous generations:

I could imagine our lived experience right now during COVID would be something like the Exclusion Act and head tax or something like that, right? Not to that extent, not even close, of course! But just think about it from that [perspective] and then you look at why people are protective, why people are conservative. Because they have . . . there is something that reinforces that kind of “Okay, trust is an issue.”

Indeed, after bemoaning Chinatown’s factionalism, Alan offered this thought: “ultimately what’s amazing, though, is we can all meet and have a stake in a place called Chinatown. We do. It’s amazing. . . . It’s a place in decline [but] it still has this amazing ability to bring all these fractious groups together.”

As predicted by cultural trauma theory, accounts of past harm allow groups to extend feelings of solidarity across time and social divisions. Other NNFs, by contrast, frequently generated disagreement. For example, some participants celebrated Chinatown’s role as a tourist destination, but others did not. “I prefer Chinatown to die than . . . to turn into an amusement park” said Teresa, the Canadian-born artist quoted earlier. Likewise, some groups embraced the traditional image of Chinatown as a refuge for working-class immigrants “arriving with energy, as well as fresh hopes and aspirations” (Chicago Metropolitan Agency for Planning 2015:10). Others dismissed this idea as dated, including recent professional-class immigrants who did not identify with “those people from Hong Kong, long ago, [who came] here with a dollar in their pocket [just trying] to survive” (Julia, a woman in her mid-40s who immigrated from China). When it came to cultural trauma, however, while participants emphasized different events, and a large minority did not use trauma narratives at all, we found no evidence of people who explicitly disagreed that Chinatowns were shaped by a history of anti-Chinese racism.

Sociologists often seek to explain variation, but understanding consistency is also important (Lieberson 1987:99–107). In this case, the ubiquity and consensus around trauma narratives in Chinatown demands explanation. Accepting the constructivist view that harmful events in the past do not in themselves guarantee that representations of these events will continue to play an important role in the present, we must explain why these narratives resonate with Chinatown activists. In answering this question, we came to understand the role of the built environment as a medium connecting past episodes of harm with contemporary processes of commemoration.

## Trauma Icons

Sociologists have long argued that collective memory is shaped by some combination of past events and the biases and interests of subsequent generations who actively construct and deploy historical narratives (Schwartz 1982; Simko 2020), yet scholars are often more interested in the latter. The past is usually seen as passively constraining memory, as it “is not infinitely malleable” through creative interpretation (Simko 2012:893). In examining trauma narratives in Chinatown, we came to a different understanding of the past: as something that actively lingered in the present, primarily through physical traces it had deposited into the physical environment. Trauma narratives were rarely discussed in the abstract; they were usually connected to specific locations and objects. Participants frequently mentioned buildings or streets while recounting larger stories of harm. Or, they might begin by discussing the closure of a restaurant before segueing into a broader point about Chinese history.

These objects and places served as “iconic” representations of trauma, providing community members with tangible evidence of the past, visible to anyone in Chinatown. “Iconicity” refers to the “ability [of objects] to articulate and intensify cultural meanings, narratives and myths into an aesthetically striking, easily recognizable, emotionally rich and symbolically relevant visual-material entity” (Solaroli 2015:1). Such icons allow people to feel “a sense of participation in something fundamental” (Bartmanski and Alexander 2012:2). Some of these icons were purposely designed memorials of the type mentioned earlier, but most were of a different sort: mundane spaces such as office buildings or restaurants that evoked memories of past harm. The way these buildings connected to the past also varied considerably.

Making sense of this variation, we identify three types of iconic objects that were important in the articulation of trauma narratives: “stubborn,” “entropic,” and “remedial.” These icons are distinguished by their material qualities, including the process through which they connect the past to the present, and the ways they fit into the lived experience of contemporary generations. All three types act as cultural “prompts” that shape processes of collective meaning-making in Chinatown (Lizardo 2016:202).

### Stubborn Icons

Most studies of objects and the built environment emphasize their “obduracy” (Bartram 2015; Gieryn 2002; Mukerji 2010). As Molotch (2003:11) writes, “objects work to hold meanings more or less still, solid, and accessible. . . . They form the tangible basis of a world that people can take to be a world in common.” Chinatowns are filled with such objects, which have remained stable across generations. Sociologist Kwok Bun Chan (1986:70) described how the “gigantic, large-scale, ultra-modern federal and provincial government buildings hover over and encroach upon the low-lying buildings of Chinatown like tombstones, seemingly reminding the Chinese people that the days of their urban space are numbered.” He was referring primarily to the newly-built Guy Favreau federal building that occupies six acres of what used to be part of Montreal’s Chinatown. It is one of a series of major postwar development projects that now surround Montreal’s Chinatown, hemming it in on all sides. Montreal is a particularly vivid example of how major urban renewal projects have dramatically shaped the geography of Chinatowns (Vitiello and Blickenderfer 2020). These projects have remained fixed in the landscape for decades, serving as conspicuous reminders of the past for future generations.

In Calgary, the Harry Hays federal building was completed in 1979, replacing two blocks of houses in Chinatown. Since then, its hulking brown exterior has marked the eastern boundary of the neighborhood. More than 40 years later, it still angers the community, many of whom were not born yet or were not old enough to remember its original construction. Dale Lee Kwong, a Canadian-born writer and self-described “Chinatown advocate” who used to lead walking tours of the neighborhood, expressed her dislike of the building:

I’m really livid about the Harry Hays building, even now! People don’t know that it’s technically part of Chinatown. To me it’s just an eyesore. And this thing they did should be acknowledged. They should have a plaque that says: more than 70 intergenerational families were living here together, and we kicked them all out and we relocated them! Imagine what Calgary’s Chinatown would be like today if that land had not been appropriated!

Harry Hays is only one of several massive office buildings that were constructed around the edges of Calgary’s Chinatown in the subsequent decades, most of which were designed with their “back of house” features facing Chinatown, including loading docks, fire escapes, and exhaust vents. Nonetheless, Harry Hays is typically the building people mention when describing this trend. Cynthia, who immigrated to Canada from China in the 2000s, casually referenced Harry Hays while describing the idea of a shrinking Chinatown:

Why do I say Chinatown is shrinking? Because a few decades ago there were nine blocks in Chinatown, all of which were Chinese. Now there are only about three and a half blocks, and [another] block was almost to become a high-rise building. You see, the *Harry Hays* building used to belong to Chinatown, and many buildings used to belong to Chinatown, but they were eaten away and high-rise buildings were built. [Translated from Mandarin]

In addition to representing past harm, Harry Hays serves as a cautionary tale. In the quote above, Cynthia mentions a proposed high-rise condominium that would occupy a large parking lot in the middle of Chinatown. Viewed through the lens of Harry Hays, this proposal is seen as another assault on the community.

The experience of stubborn icons is not just symbolic. Stubborn icons contributed to a landscape that was described as “unpleasant,” “hostile,” and “dangerous.” In Calgary, a woman lamented the death of local seniors trying to cross a busy arterial street that runs through the center of the neighborhood. In Chicago, a man speculated that the Stevenson and Dan Ryan Expressways, built through the neighborhood in the 1950s, made Chinatown an attractive target for muggings and robberies. In Boston, a 1970 report accused the nearby New England Medical Center of creating a “reservoir of hostility” through its “aggressive acquisition of Chinatown property and its indifference to the health needs of those in its immediately surrounding area” (Sullivan and Hatch 1970:66). In multiple Chinatowns, people described the unpleasantness of walking through dimly lit underpasses. These experiences contributed to a general sense of fear and vulnerability that was evident in our interviews and a major focus of many Chinatown plans.

As demonstrated in the previous section, participants routinely used trauma narratives to connect present fears with historic harms, yet not everyone who described a hostile environment made an explicit link to trauma narratives. Nonetheless, sociologists of culture have demonstrated how narratives resonate when they connect to embodied emotional states (McDonnell et al. 2017:4)—a phenomenon Winchester (2016) calls “embodied metaphor.” Newcomers to Chinatown need not know the history of urban renewal or displacement to develop negative feelings toward an expressway or the blank façade of a hospital or convention center. Cities communicate, through their built form, which groups matter and which do not (Benediktsson 2022). Trauma narratives provide explicit words that describe and validate these feelings, connecting them to broader, more profound social meanings.

Not all “stubborn” traces of past harms come to symbolize that harm specifically. One block away from Harry Hays in Calgary is the Canton Block: a much smaller, two-story brick building that houses commercial units on its main floor and community associations above. Overcoming community opposition, Chinese entrepreneurs built the Canton Block between 1910 and 1911 following their eviction from Calgary’s second Chinatown. Today it still sits at the foot of the Centre Street Bridge, the most prominent entrance to Calgary’s downtown core. Its small stature stands out amid the surrounding high-rises. Having survived the exclusion and urban renewal eras, it acts as a counter-icon to Harry Hays: a product of displacement, but also a symbol of Chinatown’s success in resisting further displacement for more than a century. As with Harry Hays, references to the Canton Block were frequently incorporated into discussions of past harms. A community historian, Fung Ling Feimo (馬鳳齡), put it this way:

[Chinatown represents the] cultural past of the early Chinese settlers. Because it was built around the time of segregation when people had to live there and they weren’t allowed to live anywhere else. It represents that past. Like the *Canton Block*: those people that had nowhere to go when the railroad was completed. They were told there is nothing here for you and you must go. So, they built Chinatown as an enclave to live in a relatively safe environment.

### Entropic Icons

Harry Hays and Canton Block connect to the past through their obduracy. Other objects become iconic for the opposite reason: because they change. Sociologists of materiality have challenged the one-sided view that objects are an inert, stabilizing force in social life (Benediktsson 2022; McDonnell 2016; Rubio 2014). Instead, this work examines the influence of objects’ physical instability. “Cultural entropy” refers to how meanings become obscured as the physical media that carry them disintegrate (McDonnell 2016). Greenland (2021) applies this concept to iconicity, demonstrating how material instability can lead to the de-consecration of formerly iconic structures. When it comes to trauma, however, highly visible signs of entropy or destruction can do the opposite: turn formerly overlooked objects into powerful symbols of loss or harm.

To justify expulsion and relocation efforts aimed at Chinese people, public authorities have historically promoted the idea of Chinatown as a place of physical and moral decline (Anderson 1991). However, this narrative is not only imposed from the outside. Today, Chinatown communities themselves are deeply concerned with what they see as the decline and disappearance of formerly beloved parts of their neighborhoods, particularly as the generation who led the 1980s and 1990s revitalization era have entered retirement. The physical degradation of the public realm is a frequently-mentioned problem in Chinatown neighborhood plans. Our interview participants were similarly distressed by this and often spoke at length about restaurants and businesses that had shut down, or streets and parks falling into disrepair. As with hostile expressways and office complexes, dilapidated architecture and shuttered businesses create opportunities for community members to make connections between the present and historic harms.

Chloe (pseudonym) is a Calgary-born Chinese woman in her 30s who regularly visited Chinatown in her childhood before drifting away as a teenager. After spending time abroad for university, she returned to Calgary and to Chinatown as an adult. She provided a vivid account of what she perceived to be the neighborhood’s decline:

Interviewer: Can you tell us about what Chinatown was like [during your childhood]?

Chloe: It was really vibrant. I remember the number of banquets and large restaurants that used to be opened. Grand Isle. Treasures of China. We used to go there a lot for banquets. This is around when the Cultural Centre was just built. . . . The bakeries were very vibrant. I used to get pork, and then there used to be tons of wonton restaurants. I would say it was very vibrant. That’s where you met everyone. That’s where you’d go every weekend.

Interviewer: Are there specific places that stand out as being particularly important to your childhood?

Chloe: I would say it’s the restaurants. When I think about it now where we used to go eat *juk* (粥) or congee. I think of that place always being there. The places that have closed down from my childhood are the recent bakery, the one that used to sell those rice bowls; what are called *boot jai goh* (砵仔糕). And Chong Fat, which just closed down . . .

Interviewer: When did you first start noticing that it was changing?

Chloe: It would have to be in my teens because we stopped going down there. With the closure of Grand Isle, Treasures of China, there was just less to do.

Interviewer: What was it like coming back after all that time?

Chloe: It was great to see there were still some iconic places that we all still remember, but it was sad. Because you could see how it wasn’t being taken care of anymore. There wasn’t that level of care and desire to attract families and individuals back. And that was hard to watch.

On their own, Chloe’s feelings of sadness for a disappearing Chinatown could be described as nostalgia rather than cultural trauma. But she did not leave things there. She connected the vanishing Chinatown of her childhood to the larger history of Chinese Canadians, and their struggle for survival:

I think that a large reason why there has been a decline in Chinatowns, not just in Calgary, but across them all, is this generational divide between what it used to be and what it was for, and what it could be now. I think when Chinatowns were first built, they were meant for survival. There was a lot of manufacturing. That manufactured experience is what kind of made it. The Chinatown that was built back then was not built for you to have a family. The very first construction was built for bachelors to survive. And that worked. And then it [became] an attraction. . . . [But] Chinese Canadian identity changed. I think what’s interesting now is there is a spark for understanding what the Chinese Canadian and even Asian Canadian identity is right now. There needs to be space for that too.

Later in the interview, Chloe connected this generational shift directly to trauma:

There is a lot of trauma in the community that has to be grappled with. That comes with any community that has faced racism, discrimination. I think there’s a generation that has not resolved that trauma, and that’s okay. But there is another generation, that’s younger, that’s ready to face and tackle that trauma.

Chloe is voicing what Brown-Saracino (2021) calls “critical nostalgia,” where the shared feeling of loss can become the basis for collective solidarity and identity without a desire to recreate the past. This is a common sentiment expressed in Chinatown, particularly among younger participants regarding what Chloe called the “manufactured experience,” describing a particular consumer culture developed by Chinatown-based entrepreneurs in the twentieth century to appeal to the tastes of the White majority.

Along with Chinese American/Canadian cuisine, one of the most recognizable symbols of this consumer culture is a distinct vernacular architecture, originally developed in San Francisco’s Chinatown. As documented by architectural historian Philip Choy (2012), after Chinatown was largely destroyed during the 1906 San Francisco earthquake, Chinese entrepreneurs hired U.S. architects to rebuild the neighborhood as an exotic and inoffensive tourist destination. This was done, in part, to head off efforts by the local government to relocate the Chinese community to the edge of town. Throughout the twentieth century, Chinatowns across the United States and Canada have undertaken similar campaigns of using tourism and vernacular architecture to improve their public image and create a sustainable economic base (Light 1974; Lin 2010). The style arguably reached its peak in the late-twentieth century, coinciding with Chinatowns’ revitalization period and the rise of “fantasy city” urbanization strategies aimed at attracting tourists into city centers with playful, postmodern architecture and theme park–inspired consumer experiences (Hannigan 1998).

For some, this tourist-focused architecture represents a celebration of Chinese culture and an immigrant success story. Conversely, critics have likened it to Disneyland (Lin 2010:223; Pang and Rath 2007:197) or described it as “self-orientalizing” (Umbach and Wishnoff 2008). However, as many of these twentieth-century vernacular buildings fall into disrepair, they have taken on new significance. The former Chinese Community & Cultural Center in Philadelphia, for example, closed in 2007 and has been unoccupied since. Its ornate façade was once an “object of scorn” among young Asian American activists in the 1970s who saw it as prioritizing tourists over locals (Wilson 2015:89). Today, however, the community is fighting to save it from demolition and convert it back into a public space.

In Toronto, the shuttered Bright Pearl Restaurant became a symbol of displacement and gentrification in 2018 after the owner tore down its vernacular façade and redeveloped it into a modernist office building. The change generated significant media attention, as well as protests and vandalism. The event was a catalyst for the eventual founding of the anti-gentrification group Friends of Chinatown Toronto (FOCT) (Zheng 2021:48). A subsequent report supported by FOCT, “Community Power for Anti-Displacement,” featured a drawing of Bright Pearl’s façade on its cover page (Ahmed et al. 2020). Inside, the report includes pictures from before and after the renovation and notes that this style of architecture was “strategically designed to appeal to tourists . . . often in response to being stigmatized by the white, middle and upper class consumers” (p. 11). While lamenting the loss of the façade, described as “a disrespectful reminder of the nature of change,” the report argues that “vague Sino-architectural form does not a Chinatown make” (p. 12). In other words, the building’s value is not the architecture itself. Rather, its physical destruction symbolizes ongoing harm to the community. The report relates this to ongoing gentrification, and the historic connection between this architectural style and past struggles against stigma and discrimination.

### Remedial Icons

Past harms like urban renewal projects not only produce obdurate “tombstones” like Harry Hays or Guy Favreau: they also leave a landscape strewn with detritus, including empty and unkept lots, ruined or isolated buildings, blank walls, and areas marked by pollution or contamination. An empty lot is not particularly obdurate (it can be developed) or subject to entropy (it is already broken-down). Instead, such lots are best described as “liminal” in the sense of being a “no-man’s-land,” dislocated from community life and social order (Zukin 1991:269). Other traces are more superficial, like names embedded in the landscape of prominent locals who were involved in perpetrating past harms. Be it an abandoned lot or a park named after an anti-Chinese campaigner, these kinds of traces can be offensive or unpleasant to affected communities, motivating them to remedy the situation. In doing so, such actions offer opportunities to create new trauma icons: purposely designed memorials that address the harms of the past physically and symbolically. The production of these “remedial icons” constitutes a form of “working through” cultural trauma in a very material sense.

The Vine Street Expressway (VSE) that runs through Philadelphia’s Chinatown is illustrative, in part because most Chinatowns have faced similar expressway projects (although not all were completed). The VSE is a stubborn icon that cuts Chinatown’s main core from its north, and its construction left a trail of detritus in the form of empty lots, many surrounded by chain-link fence and barbed wire. Unlike the expressway itself, these lots are not so obdurate. Over time, Philadelphia’s Chinatown community has attempted to use some of this space to reverse, or at least mitigate, the impact of the expressway.

Friar Thomas Betz arrived at the Holy Redeemer Chinese Catholic Church in 1991 just as the VSE was being completed after a multidecade construction process. During construction, the Philadelphia Chinatown Development Corporation (PCDC) had successfully campaigned to save the church from demolition, although it remains marooned on the other side of the VSE. Betz described what it was like to arrive in Chinatown at this moment:

Our Chinatown was impacted by a big development project which was building up a 13-lane highway between Chinatown and our church and school. . . . When I came to Philadelphia, our neighborhood around the Catholic Church and school was completely blighted. So, I joined the Board of PCDC, in part to try to work with the community to try to ameliorate some of the effects of that horrible developmental program.

The liminal space surrounding the VSE became a focal point for the work of the PCDC and their allies. Since then, they have used some of this land to produce major public spaces such as the 10th Street Plaza and the Crane community center. Currently, they are working on their most ambitious project: “The Chinatown Stitch.” The Stitch will not remove the VSE, but it will attempt to hide it under a deck that will serve as a public park. Supporters of the project have adopted trauma narratives, describing the VSE as “a noose—preventing expansion and growth according to the needs of the Chinese community” (Creatura n.d.). By contrast, the Stitch is promoted as a means of healing Chinatown by “sew[ing] the disconnected parts of Chinatown together and address[ing] the ongoing harms of the Vine Street Expressway” (City of Philadelphia 2023:3). The project had received $207 million in federal funding under the Biden administration, but this was almost all cut by the subsequent Trump administration.

A similar expressway was proposed in Calgary’s Chinatown in the 1960s. In response, community leaders formed the Sien Lok Society, which pressured the municipality into canceling the project. The unbuilt expressway left behind vacant land, including an empty lot at the foot of the Centre Street Bridge, kitty-corner to the Canton Block. Following their victory against the expressway, the Sien Lok Society raised money to fund the lot’s redevelopment into a public park. Today, Sien Lok Park is filled with public art and plaques that memorialize Chinatown’s victory against the expressway, situating it within the larger narrative of hardship and overcoming among Chinese Canadians.

The Stitch and Sien Lok Park are permanent, large-scale forms of placemaking requiring substantial government support. But Chinatowns are also filled with many smaller, more informal remedial icons. In Toronto, activists associated with FOCT rebuilt a crumbling plaza in front of the Chinatown Centre Mall (an entropic icon) into the “Anti-Displacement Garden,” which has become a hub for their activism. In Calgary, Toronto-based artist Annie Wong created “a park without a name,” an art installation marking the renaming of a local park. The park had been named for James Short (1862 to 1942), a local resident and prominent educator who opposed the construction of the Canton Block in 1910 and subsequent expansions of Chinatown. Wong’s 2022 installation, which was commissioned by the City of Calgary as part of a larger Chinatown public art campaign, featured banners hung from lampposts with quotations in Chinese and English from interviews with community members, including “James Short was a racist” and “I am tired of hearing about James Short. How come this White man is talking so much?” (City of Calgary n.d.). The artwork was controversial, even before it was installed. Wong and the city faced pushback from some in Chinatown who thought it was too negative and might provoke violence amid the anti-Chinese climate of the COVID-19 pandemic. After months of delays, the installation lasted only four days. It was eventually replaced by a plaque that used more neutral language, noting that the park (now called 和園 Harmony Park) was renamed “to address the historic harm caused by racial discrimination against the Chinese Community.” No mention is made of James Short.

The short-lived exhibition illustrates how remedial icons can vary considerably in their scope and longevity, often depending on the level of consensus surrounding the initiative and the financial and political resources available to proponents. On this issue, we return to familiar territory in existing constructivist research on memorial entrepreneurship that demonstrates how contemporary biases, interests, and power dynamics influence commemoration (Jordan 2006; Wagner-Pacifici and Schwartz 1991). Also consistent with this research is that placemaking projects themselves become opportunities for the public articulation of trauma narratives. We observed how community members invited historians to speak publicly or advise on projects, or how older activists chided city officials for a decades-long history of insensitivity to Chinatown. Generally overlooked in existing constructivist accounts, however, is the role that historic events themselves play in leaving behind places and objects that subsequent generations feel compelled to address in the first place.

Once completed, remedial icons become additional pieces of the urban environment that shape the experience of cultural trauma. Unlike stubborn or entropic icons, remedial icons are more than just reminders of past harm: they often serve as resources in ongoing struggles against present or future harm. This became evident during the COVID-19 pandemic when anti-Chinese sentiment and violence surged (Chen and Wu 2025). Chinatowns were uniquely vulnerable to the effects of the pandemic, but they were also the location of many anti-racist rallies during this period, including during the 2021 Stop Asian Hate movement (Patterson 2026). Spaces like Sien Lok Park and the Anti-Displacement Garden serve as symbolically-charged spaces for mobilizing resistance.

Remedial icons are also media for communicating trauma narratives and extending sympathies to new groups and generations, through plaques, artwork, or exhibitions. Tony Wong, an immigrant from Hong Kong in his 60s, is the current president of the Chinese Cultural Centre in Calgary Chinatown. He shared his hope that the Centre, and the history it preserves, could act as a bulwark against contemporary anti-Chinese racism:

When Donald Trump was president, he once talked about the “Wuhan Virus” and that the “Chinese are the problem of our employment.” I just hope that people who have visited the [Chinese Cultural Centre] will have sympathy for us after hearing our stories. They will know that the head tax and Chinese exclusion act hurt us very much.

## Discussion: Chinatown as a Cultural Reservoir

Beginning this research, we were confounded by a seemingly simple question. What *is* Chinatown for the people so passionately fighting for its preservation? Existing sociological definitions were unsatisfactory. Chinatown was not an “ethnic enclave” in the traditional, Chicago School sense of a stopping point for immigrants on the path to cultural assimilation or socioeconomic mobility, or even more recent definitions as a networking and transportation hub for new immigrants (Liang et al. 2018). Quite the opposite: placemakers were more likely to be established immigrants or Canadian/U.S.-born living elsewhere and looking to give back to the community. Chinatown was not a “cultural community,” defined as a boundaryless collection of ethnic amenities like language schools, churches, or grocery stores (Ling 2005). Placemakers are quite concerned with Chinatown’s boundaries, even as Chinese amenities have become widely available outside the neighborhood (Fong et al. 2007; W. Li 1998). Nor was Chinatown primarily an “enclave economy” (Zhou and Logan 1989). Many participants had financial ties to the neighborhood through employment or business-ownership, but few discussed their motivations in economic terms. These definitions fit some Chinatowns and some groups better than others, but no definition provided a satisfying explanation for the participants in this project. In part, this is because these models frame Chinatown primarily as a means to an end. Our participants saw it as an end in itself.

Another way to think about Chinatown is as a “place” (Molotch et al. 2000) or “scene” (Silver and Clark 2016): a geographically-bounded location with a unique cluster of objects, people, and experiences invested with cultural meaning. These concepts have clear connections to the data presented above. On the other hand, while all places are invested with meaning, few demonstrate the level of “cultural power” or “resonance” of Chinatown, which provokes “heightened emotions [and] remind people of their moral commitments to the group” (McDonnell 2016:30).

Our team began using the term “cultural reservoir” to describe Chinatowns, attempting to capture the deep accumulation of meaning that fills these neighborhoods, covering their streetcorners, empty storefronts, popular restaurants, high-rise office blocks, and parks and plazas. Chinatowns provide participants with “a sense of participation in something fundamental” (Bartmanski and Alexander 2012:2). This feeling was evident in how some contrasted themselves with “outsiders” who did not truly “know” Chinatown:

If you don’t have a personal connection with it, you’ll see Chinatown as a commercial shopping center. . . . A White or non-Chinese person, or even a Chinese person who hasn’t seen the value of this area—that may not know what Chinatown is all about—wouldn’t fight for it because they only know Chinatown from the outside. (Alice, Canadian-born woman in Calgary, 30s)

To me a house is a place where you sleep and wake up in the morning. Home is Chinatown. . . . For me, Chinatown gave me the confidence to protect my Chinese American identity, and I think that people who grew up in the suburbs don’t have this cultural confidence. (Mark [pseudonym] American-born man in Philadelphia, 50s)

Notably, neither of these participants lives in Chinatown. Nonetheless, they felt “summoned” by a sense of collective responsibility connected to the neighborhood (Tavory 2016).^4^

By analyzing the NNFs (neighborhood narrative frames) used by participants and incorporated into planning documents, we were able to uncover the dominant meanings that “fill” the reservoir. Trauma narratives stood out in their ubiquity, prominence, and degree of consensus, cutting across groups who disagreed on many other issues. In fighting to preserve Chinatown in the twenty-first century, our participants felt part of a shared enterprise with previous generations, including anti–urban renewal activists of the 1960s and “bachelors” living under exclusion laws.

Thinking of Chinatown as a “cultural reservoir” helps adjudicate the larger debate over whether experiences of trauma are produced by historic events themselves, or by the cultural work of “memorial entrepreneurs” who create and popularize trauma narratives. In line with constructivism, our participants are “carriers” of trauma narratives, relying on their “agency, creativity, and imagination” (Simko 2020:72) to articulate and mobilize these narratives in public meetings, when authoring planning documents, and by designing placemaking projects. In line with realism, they do this within a “figured world,” physically shaped by the very events they seek to commemorate, in which certain narratives appear “inevitable, natural or true” (Mukerji 2010:404). According to Mukerji (2010:404), physical landscapes are a conduit of power, through which the regimes of the past “shape the conditions of possibility for collective life” for subsequent generations. Chinatown activists today are still locked in a struggle with the elites of yesterday who demolished homes to build expressways or, earlier, pushed Chinese residents and businesses into segregated neighborhoods in the first place. However, while Mukerji describes a figured landscape as something that people take for granted, we have seen how Chinatown activists use cultural trauma to problematize their neighborhood. Noisy expressways, imposing hospitals, and shuttered businesses are interpreted not as inevitable parts of inner-city living, but as harms inflicted upon them in a generations-long campaign of Chinese exclusion.

While more specific than “place,” cultural reservoirs are more diffuse than memorial sites, which are usually sacred spaces set aside from everyday life. Reservoirs may contain such memorials, but those are only one of many features that make historical events relevant to contemporary communities. Within reservoirs, the sacred intermingles with the profane. Memories of existential harm are evoked by a wide variety of iconic objects that are often encountered and experienced within the mundane course of everyday life. These objects have been deposited there through multiple processes, which we grouped into “stubborn,” “entropic,” and “remedial” icons.

The icons that fill the reservoir are not culturally neutral; they have “affordances” (Benediktsson 2022; McDonnell 2016:27). Their direct connection to the past and their aesthetic qualities connect more readily to some narratives than others. In Chinatowns, icons share “mnemonic relatedness” (Suttles 1984:294) that allows people to interpret a common set of meanings from spaces as physically different as Harry Hays, Canton Block, and Sien Lok Park. This coherence exists, in part, because Chinese Canadian and U.S. history has its own coherence. From the Exclusion era to urban renewal to the recent COVID-19 pandemic, Chinese Canadians/Americans have often been treated as unwelcome guests—the targets of “nativistic racism” (Kim 2007). This does not mean that collective memory in Chinatown is a clear, unambiguous snapshot of the past. People can and do talk about Chinatown through alternative narratives. Rather, to borrow evolutionary language from Abrutyn (2015), a history of exclusion and displacement has created conditions that “select” for trauma narratives as communities attempt to understand and respond to the reality that confronts them.

Trauma icons structure memory by “serv[ing] as prompts for persons to engage in (individual and collective) acts of meaning construction” (Lizardo 2016:202). When Cynthia (quoted earlier) references Harry Hays, she is appealing to a piece of reality that is shared not only between her and the interviewer, but also with the families displaced in the 1970s. In this sense, icons are powerful “meaning maintainers” that provide “scaffolding” for cognition and communication (Taylor, Stoltz, and McDonnell 2019), enabling the reproduction of trauma narratives between people and over time.

Finally, more than just symbolic reminders, cultural reservoirs are a material medium through which people interact with the past. Placemakers work on the landscape given to them by history and, in doing so, they are guided by collective memory. Remedial icons demonstrate how this process can be used by communities to “work through” trauma by physically transforming offensive traces of past harm into places that facilitate collective action, memory, and the extending of sympathies beyond immediate victims. In this sense, remedial icons are like healed “scars” that create permanent reminders but no longer generate harm (Simko and Olick 2020:656).

Not all wounds are easily healed, however. Simko (2020) distinguishes “working through” versus “acting out” trauma. The latter refers to a situation in which communities become stuck in a cycle of re-traumatization and distrust. Stubborn icons, by definition, resist physical change. The Vine Street Expressway, for example, continues to create pollution and mobility challenges in Philadelphia’s Chinatown. Efforts to remediate it have required unprecedented financial and political investment, and progress can be undone by a single election. Most Chinatowns have little hope of transforming stubborn icons. Even entropic icons often crumble into detritus before they can be saved. Finally, the controversy over “a park without a name,” which some feared would provoke the very anti-Chinese sentiment it sought to address, demonstrates that remedial icons themselves can sow distrust. In summary, the unique physical makeup of a cultural reservoir greatly influences the nature of the trauma process and its outcome.

## Conclusions

Let us return to Kamloops, British Columbia, in 1977, where Jim Wong-Chu walked among the fading graves in the old Chinese cemetery, “searching for scraps of memory.” Traveling three miles up the South Thompson River from the cemetery, we arrive at the Kamloops Indian Residential School located in the Tk’emlúps te Secwépemc First Nation, then in its final year of operation. Now let us move forward to the year 2021, more than four decades after the school’s closure, and six years after the Truth and Reconciliation Commission of Canada (2015:1) declared Indian Residential Schools (IRS) a weapon of “cultural genocide.” At this time, in this place, the Tk’emlúps te Secwépemc First Nation announced that ground-penetrating radar had detected evidence of what they believed to be hundreds of unmarked graves on school grounds (Dickson and Watson 2021). The harms of the IRS had been documented and widely reported years earlier, but the sudden appearance of physical evidence sparked widespread public grief among Indigenous and non-Indigenous people across Canada. This response dwarfed initial reactions to the Truth and Reconciliation report. In their grief, some protesters destroyed iconic representations of those seen as responsible for the IRS, setting fire to churches and toppling statues.

The commemoration of cultural trauma is necessarily shaped by the way physical traces of past events disappear, reappear, transform, or remain stubbornly situated in the environment. As such, the material landscape serves as an important medium connecting past harms to the contemporary “trauma process.” The concepts of “trauma icons” and “cultural reservoirs” introduced in this article advance understandings of cultural trauma by providing a non-determinative understanding of the role of past events in shaping collective memory, and they open new avenues for research within cultural trauma studies and other fields.

This study focused on commonality, but it is also important to look for variation in how trauma is experienced. By comparing the resonance of trauma narratives across different groups, we can gauge the relative influences of the physical environment versus the characteristics of “carrier” groups (Simko 2020). For example, previous studies have demonstrated that people’s perceptions of place are influenced by the spatial practices that bring them into a given location (Patterson 2016). How might residents, employees, or tourists differ in their perceptions and interpretations of trauma icons compared to the placemakers studied here? Alternatively, we can compare demographically similar groups who do and do not have close contact with a cultural reservoir, such as Chinatown communities compared to Chinese people living in ethnoburbs or outside enclaves altogether.

Cultural reservoirs also vary. Chinatowns differ in significant ways, for example, between those that successfully defeated expressway projects (e.g., Calgary, Toronto) and those that did not (e.g., Philadelphia, Montreal). How might the unique assemblage of icons within a given neighborhood influence the content and salience of trauma narratives? These insights can also be expanded to the literature on place branding and entrepreneurship. To what extent does the content of cultural reservoirs constrain and shape the work of memory entrepreneurs? Here the “mnemonic relatedness” of Chinatowns contrasts with other historic neighborhoods with more varied pasts. Hunter, Loughran, and Fine (2018), for example, document postwar efforts to promote Philadelphia’s Independence Hall area as the “birthplace” of American democracy. This narrative was at odds, however, with the area’s subsequent history as a home of segregated communities of color. Rather than revising their narrative to incorporate these communities, the entrepreneurs tried (ultimately unsuccessfully) to physically erase the communities by encouraging gentrification and displacement. The approach developed here can help us situate these “memory politics” (Hunter et al. 2018) within and toward physical landscapes shaped by various material processes that have created cultural “affordances.”

In the study of race and ethnicity, cultural reservoirs provide an alternative way of thinking about the spatialization of ethnicity beyond an “enclave epistemology” tied to migration and assimilation (Ghaziani 2019). As the Asian American population has grown, diversified, and dispersed, Tran (2024:583) has advocated replacing a unidimensional focus on assimilation/racialization with an “intra-Asian diversity lens” that examines factors driving convergence or divergence among groups. More generally, cognitive approaches to ethnicity have focused on how schema and narratives shape feelings of “groupness” (Brubaker, Loveman, and Stamatov 2004). Cultural reservoirs demonstrate how the urban landscape influences this process and, by extension, feelings of sympathy and identification among co-ethnics and across ethnic boundaries. As we have seen in Chinatowns, trauma narratives often (but not always) bridge otherwise “fractious” ethnic and generational divides. Because trauma is a common experience among racialized communities (Asian and non-Asian) (Nagata et al. 2024), we can examine how its material traces influence cross-ethnic identification or differentiation. A good place to start might be to compare Chinatowns with Little Saigons, which have their own collections of trauma icons related specifically to the Vietnam War and the refugee experience (Allen-Kim 2024; Nguyen 2018). How do these unique histories of trauma, and the way they materialize in the urban landscape, influence mutual identification between Chinese and Vietnamese Americans/Canadians and beyond?

Finally, this article can help advance the sociological study of place more generally. Bell (1997:813) once argued that the phenomenology of place often includes a “sense of the presence of those who are not physically there,” which he called the “ghosts of place.” His term captures how certain locations, like Chinatowns, stand out as being particularly resonant and generate feelings of profound connection to others, alive or dead. More recently, sociologists such as Silver and Clark (2016) have sought to advance the phenomenology of place by connecting subjective experiences to systematically measurable features of the urban landscape. The perspective developed here supports this direction by identifying the material foundation that gives rise to the “ghosts of place,” helping us understand how the past can become a sacred presence lurking within people’s otherwise profane, everyday lives.

As we write this, U.S. cities are witnessing ongoing, and often violent, immigration raids reminiscent of the efforts to expel Chinese migrants during the Exclusion era. The raids are erasing people, communities, and experiences from the urban landscape, but leaving behind traces of their presence, from damaged or emptied buildings to sprawling detention centers. As fragmented, hyperpartisan media ecosystems undermine mutual empathy and a sense of shared reality (Finkel et al. 2020), these physical traces and the “ghosts” that inhabit them will be important tools for any possibility of civil repair and rebuilding feelings of solidarity.

## Supplemental Material

sj-pdf-1-asr-10.1177_00031224261422414 – Supplemental material for Living with Ghosts: How Physical Traces of the Past Shape Cultural Trauma in Chinatowns Supplemental material, sj-pdf-1-asr-10.1177_00031224261422414 for Living with Ghosts: How Physical Traces of the Past Shape Cultural Trauma in Chinatowns by Matt Patterson, Henry Tsang, Bryan Kuk, Weiqi Li and Mojtaba Rostami in American Sociological Review

## Notes

1. Yu (2001) provides a thorough account of the relationship between the Chicago School and Asian Americans, including the work of these scholars.

2. Cantonese speakers represent a plurality of our participants, but most were multilingual and requested an English-language interview.

3. Similar displacement narratives exist in Chicago and Toronto, among other cities.

4. Tavory (2016) introduced the concept of “summoning” to describe the feelings of obligation among Orthodox Jews living in a Jewish enclave. Although the populations and context are different, the term’s religious connotations fit the sense that Chinatown is continually “summoning” community members to take up the fight against Chinese exclusion.
