# Supplementary material for: Living with Ghosts: How Physical Traces of the Past Shape Cultural Trauma in Chinatowns
Source: Am Sociol Rev. 2026 Mar 13;91(2):286–312. doi: 10.1177/00031224261422414 (PMC13068313; doi:10.1177/00031224261422414)
Supplement: sj-pdf-1-asr-10.1177_00031224261422414 – Supplemental material for Living with Ghosts: How Physical Traces of the Past Shape Cultural Trauma in Chinatowns [file sj-pdf-1-asr-10.1177_00031224261422414.pdf]

Online Supplement to:

Living with Ghosts: How Physical Traces of the Past Shape Cultural Trauma in Chinatowns

Matt Patterson
University of Calgary

Henry Tsang
Athabasca University

Bryan Kuk
University of Calgary

Weiqi Li
University of British Columbia

Mojtaba Rostami
University of Calgary

This online supplement contains our interview guide template (Section A) and a list of 83 Chinatown planning documents that we analyzed for the study (Section B). They are provided to support and encourage further research on historic Chinatowns and their role in cities today.

A. INTERVIEW GUIDE

We designed the following question to elicit neighborhood narrative frames through which our participants understood Chinatown. We used the guide as an initial template and modified it to fit each participant's unique role and type of placemaking practice.

Introduction

- We would like to begin by asking you for some basic demographic information. Could you please tell us your:
    - Age
    - Gender
    - Occupation
    - Highest degree you've earned
    - Place of birth
    - Ethnicity
    - Language Ability

Personal Experience with Chinatown

Note: these questions are designed to encourage participants to share personal stories, anecdotes, and memories about Chinatown. The intention is to understand the interrelationship between their personal identity and biography and the characteristics of Chinatown as a place.

- Do you or have you ever lived in Chinatown?
- How did you become involved with [organization, campaign, art project, business, development project, etc.]

- Tell me about your role in this [organization, campaign, etc.]
- How do you think your [organization, campaign, etc.] contributes to the Chinatown neighborhood?
- What has changed about the neighborhood in the time you've lived/worked here?
- Are there any places within Chinatown that have been particularly important in your life?
- Are there any other stories or memories that you would like to share about the time you have spent in Chinatown?

Personal Evaluation of Chinatown
Note: this section is used to elicit the participant's normative understanding of Chinatown: how they feel about the neighborhood at the present and what their goals, hopes, or fears are for the neighborhood in the future.

- How would you describe Chinatown as a neighborhood to someone who has never visited it before?
- What are the most important places within Chinatown?
- How well does Chinatown serve the needs of the community?
- What aspects of Chinatown would you like to see preserved for future generations?
- What (if anything) would you like to see changed in Chinatown?
- Are there any problems that Chinatown faces? If so, what are they?
- Are there any areas in Chinatown or places within Chinatown that bother you and that detract from the neighborhood?

Conclusion

- Do you think there are any important issues regarding Chinatown that we haven't covered in this interview? If so, what are they?
- As we continue this research, are there any questions you would like to see us investigate or information that we could find which would be useful to you and your community?
- Are there any people or organizations that you think would be important for us to talk to during our research?

B. CHINATOWN PLANNING DOCUMENTS

The following is a list of Chinatown planning documents that we analyzed for this project. The documents were published between 1970 and 2024 in 10 American and 6 Canadian cities.

Year	City	Title	Main Author
1970	Boston	The Chinese in Boston, 1970	Action for Boston Community Development
1971	Boston	Chinatown Planning Project: A Pre-Conference Study	Chinese-American Civic Association Boston; Harvard Field Service
1980	Los Angeles	Chinatown Redevelopment Plan	Community Development Agency of the City of Los Angeles
1986	Calgary	Chinatown Area Redevelopment Plan	City of Calgary (Planning and Development)

1986	Calgary	Chinatown Handbook for Public Improvements	City of Calgary (Planning and Development)
1986	Chicago	Chinatown Basin Tax Increment Redevelopment Plan	City of Chicago
1988	Boston	Chinatown 2000	Chinatown-South Cove Neighborhood Council; MIT Urban Design Studio
1990	Boston	Chinatown Community Plan: A Plan to Manage Growth	Chinatown-South Cove Neighborhood Council; Boston Redevelopment Authority
1990	Boston	Chinatown Community Plan Update	Chinatown-South Cove Neighborhood Council; Boston Redevelopment Authority
1994	Boston	The Chinatown Community Assessment Report	The Chinatown Coalition
1998	Seattle	Chinatown/International District Strategic Plan	City of Seattle
2000	Boston	Chinatown Master Plan 2000	The Chinatown Coalition; City of Boston
2001	Los Angeles	Chinatown Redevelopment Project - Amendment	Community Development Agency of the City of Los Angeles
2002	Chicago	Chinatown Basin Tax Increment Redevelopment Plan (Amendment)	City of Chicago
2002	New York City	Chinatown: One Year After 911	Asian American Federation of New York
2002	Vancouver	Chinatown Vision Report	City of Vancouver (Community Services)
2003	San Francisco	Chinatown Area Plan	San Francisco (Planning Dept)
2004	New York City	America's Chinatown: A Community Plan	Asian Americans for Equity
2004	New York City	Chinatown Access and Circulation Study	Lower Manhattan Development Corporation
2005	New York City	Chinatown Arts Center Feasibility Study	Committee to Revitalize and Enrich the Arts and Tomorrows Economy in Chinatown
2006	Los Angeles	Chinatown Industrial Area Data and Recommendations	City of Los Angeles (Planning Dept)
2008	New York City	A Cultural Heart for Chinatown	Committee to Revitalize and Enrich the Arts and Tomorrows Economy in Chinatown
2009	Edmonton	The Quarters Area Redevelopment Plan	City of Edmonton (Urban Planning and Economy Dept)
2009	Edmonton	The Quarters Urban Design Plan	City of Edmonton (Urban Planning and Economy Dept)

2009	Vancouver	Application for National Historic Site Designation	City of Vancouver (Community Services)
2009	Washington, DC	Chinatown Cultural Development Small Area Plan	District of Columbia (Office of Planning)
2010	Boston	Chinatown Master Plan 2010	Chinatown Master Plan Committee/City of Boston
2010	Los Angeles	Chinatown Redevelopment Area 5-Year Plan	Community Development Agency of the City of Los Angeles
2011	Honolulu	Chinatown Riverwalk Revitalization Connectivity Study	City and County of Honolulu (Office of Economic Development)
2011	Vancouver	Chinatown Design Policies	City of Vancouver (Planning Dept)
2011	Vancouver	Chinatown Economic Revitalization Action Plan	City of Vancouver (Planning Dept)
2012	Edmonton	The Quarters Revitalization Levy Area Plan	City of Edmonton
2012	Vancouver	Chinatown Neighborhood Plan and Economic Revitalization Strategy	City of Vancouver (Planning Dept)
2013	Chicago	Chinatown Community Vision Plan - Existing Conditions Report	Coalition for a Better Chinese American Community; Chicago Metro Agency for Planning
2013	New York City	Preserving Affordability and Authenticity	Chinatown Working Group/Pratt Center for Community Development; The Collective for Community, Culture and the Environment
2013	Philadelphia	Callowhill-Chinatown North Strategic Plan	Philadelphia City Planning Commission
2013	San Francisco	Chinatown-Broadway Street Design Report	San Francisco (Planning Dept)
2013	Toronto	East Chinatown Revitalization Strategy Report	Ryerson University, Ward 30 Councilor Office, Chinese Chamber of Commerce
2014	Portland	Old Town/Chinatown Five-Year Action Plan	Portland Development Commission
2015	Chicago	Chicago Chinatown Community Vision Plan	Coalition for a Better Chinese American Community; Chicago Metro Agency for Planning
2016	Calgary	Guiding Principles for Development in Chinatown	City of Calgary (Planning and Development)
2016	Calgary	Chinatown Business Vitatlity Study - Existing Conditions	City of Calgary; Chinatown District Business Improvement Area
2016	Edmonton	Chinatown Economic Development Plan	City of Edmonton (Planning and Development Dept)
2016	Honolulu	Chinatown Action Plan	City and County of Honolulu

2016	Los Angeles	Central City North Community Plan Area: Historical Resources Survey Report	Los Angeles City Planning
2016	San Francisco	Urban Displacement Project: San Francisco's Chinatown	UC Berkeley Urban Displacement Project
2016	Seattle	Sustainable Neighborhood Assessment: Chinatown-International District	Global Green USA
2017	Calgary	Exploring Communities Report: Chinatown	Federation of Calgary Communities; University of Calgary
2017	Calgary	Chinatown Business Vitatlity Strategy and Action Plan	City of Calgary; Chinatown District Business Improvement Area
2017	Chicago	Chinatown Anti-Displacement Community Project Report	Coalition for a Better Chinese American Community; Loyola University Chicago
2017	Edmonton	Chinatown Urban Interface Plan	City of Edmonton
2017	Edmonton	Chinatown Strategy	City of Edmonton
2017	Philadelphia	Chinatown Neighborhood Plan	Philadelphia Chinatown Development Corporation
2017	Portland	New Chinatown/Japantown Historic District Design Guidelines	Portland Bureau of Planning and Sustainability
2017	Seattle	Seattle Chinatown/International District 2020 Healthy Community Action Plan	InterIm Community Development Association
2017	Vancouver	Chinatown Food Security Report	Hua Foundation
2017	Vancouver	Retail Gentrification Mapping Report	Carnegie Community Action Project
2017	Vancouver	Historical Discrimination Against Chinese People in Vancouver	Advisory Group on Historical Discrimination Against Chinese People in Vancouver; City of Vancouver (Community Services)
2017	Washington, DC	Realizing a New Vision for Chinatown Park	Urban Land Institute Washington, City of Washington Mayor's Office
2018	Los Angeles	Chinese Americans in Los Angeles: Historical Context Statement	Los Angeles City Planning
2018	Philadelphia	People Power Place: A Cultural Plan for Chinatown North/Callowhill	Asian Arts Initiative
2018	Seattle	Chinatown/International District Plan - Status Report	City of Seattle Office of Planning and Community Development
2018	Vancouver	Chinatown Social Cohesion Report	Hua Foundation
2019	Calgary	Chinatown Historical Context Paper	City of Calgary
2019	Washington, DC	Chinatown Design Guide	District of Columbia Office of Planning

2019	Winnipeg	A Development Strategy for Northwest Exchange District and Chinatown	Centreventure Development Corporation
2020	Boston	Chinatown Master Plan 2020	Chinatown Master Plan Committee; City of Boston
2020	Chicago	Chinatown Parking Management Plan	Coalition for a Better Chinese American Community; Chicago Metro Agency for Planning
2020	Los Angeles	Downtown Community Plan: Chinatown	Los Angeles City Planning
2020	Toronto	Community Power for Anti-Displacement: An Inclusive Future for Downtown Chinatown	Friends of Chinatown TO; University of Toronto Dept. of Geography and Planning
2021	Calgary	Chinatown Sense of Place Study	City of Calgary; University of Calgary
2021	Calgary	Chinatown Mobility Study	City of Calgary; University of Calgary
2021	Montreal	Plan d'Action 2021-2026 Pour le Development du Quartier Chinois	City of Montreal
2021	New York City	Chinatown Downtown Revitalization Application	Coalition to Revitalize Chinatown
2022	Calgary	Chinatown Cultural Plan	City of Calgary
2022	New York City	Chinatown Strategic Investment Plan	New York State Dept of State
2022	Vancouver	Vancouver Chinatown Cultural Heritage Assets Management Plan	City of Vancouver (Planning Dept)
2023	Calgary	Chinatown Area Redevelopment Plan	City of Calgary (Planning and Development)
2023	Montreal	Chinatown Reimagined Montreal Forum Proceedings	JIA Foundation
2023	Philadelphia	The Chinatown Stitch: Vision Report	City of Philadelphia; Philadelphia Chinatown Development Corporation
2023	Vancouver	Chinatown Uplifting Plan	City of Vancouver
2024	Vancouver	Chinatown Cultural District Framework	City of Vancouver
